# Hypoxia-Regulated miRNAs in Human Mesenchymal Stem Cells: Exploring the Regulatory Effects in Ischemic Disorders

**DOI:** 10.3390/ijms20061340

**Published:** 2019-03-16

**Authors:** Carmela Dell’Aversana, Francesca Cuomo, Chiara Botti, Ciro Maione, Annamaria Carissimo, Amelia Casamassimi, Lucia Altucci, Gilda Cobellis

**Affiliations:** 1 Department of Precision Medicine, Università degli Studi della Campania L. Vanvitelli, Via L. De Crecchio, 7, 80138 Naples, Italy; carmela.dellaversana@unicampania.it (C.D.); francescacuomo8@gmail.com (F.C.); chiara_botti@yahoo.it (C.B.); maione.ciro@libero.it (C.M.); amelia.casamassimi@unicampania.it (A.C.); lucia.altucci@unicampania.it (L.A.); 2 Istituto per le Applicazioni del Calcolo Mauro Picone (CNR), via P. Castellino, 111, 80138 Naples, Italy; a.carissimo@iac.cnr.it

**Keywords:** homing, miRNA, hypoxia, CLI, migration, inflammation, hMSCs

## Abstract

Human mesenchymal/stromal stem cells (hMSC) are the most promising cell source for adult cell therapies in regenerative medicine. Many clinical trials have reported the use of autologous transplantation of hMSCs in several disorders, but with limited results. To exert their potential, hMSCs could exhibit efficient homing and migration toward lesion sites among other effects, but the underlying process is not clear enough. To further increase the knowledge, we studied the co-regulation between hypoxia-regulated genes and miRNAs. To this end, we investigated the miRNA expression profile of healthy hMSCs in low oxygen/nutrient conditions to mimic ischemia and compared with cells of patients suffering from critical limb ischemia (CLI). miRNAs are small, highly conserved, non-coding RNAs, skilled in the control of the target’s expression level in a fine-tuned way. After analyzing the miRNOme in CLI-derived hMSC cells and healthy controls, and intersecting the results with the mRNA expression dataset under hypoxic conditions, we identified two miRNAs potentially relevant to the disease: miR-29b as a pathological marker of the disease and miR-638 as a therapeutic target. This study yielded a deeper understanding of stem cell biology and ischemic disorders, opening new potential treatments in the future.

## 1. Introduction

Ischemic disorders are characterized by reduced blood flow to a tissue or organ due to unfavorable vascular conditions, generally caused by atherosclerosis, such as stroke, myocardial infarction, and critical limb ischemia (CLI). They share the same etiopathological conditions, and to counteract the progression of the disease, pharmaceutical and surgical approaches have been explored to restore the function of arteries, although they cannot support long-term tissue regeneration and recovery. Thus, alternative approaches have to be considered.

Among these, the autologous transplantation of bone marrow-derived mesenchymal/stromal stem cells (MSC) contributes to tissue regeneration due to their multilineage differentiation capacity, immunomodulatory and anti-inflammatory effects, capacity to secrete trophic factors that are able to induce neovascularization and inhibit cell death, as well as their migratory capacity to reach the injured sites [1].

For these reasons, MSCs have become an attractive cell system for clinical applications in different disorders, due to the feasibility of isolation, safety in transplantation, and benefits for patients irrespective of age [2].

Critical limb ischemia is a severe disorder in which the lack of blood flow can cause necrosis and gangrene in the feet and legs, ultimately resulting in limb amputation [3,4]. 

Cell-based therapy is increasingly recognized as a promising therapeutic option for patients in which the autologous transplantation of hMSCs is applied to induce angiogenesis and promote the regeneration of ischemic tissues, but with limited results [5,6]. In fact, the benefits observed in preclinical animal model studies have not been convincingly reproduced in ongoing clinical trials due to poor cell engraftment, short cell persistence after transplantation, and low cell survival rate [7]. This may be due in part to the lack of suitable human ischemic models for unequivocally assessing the cell dosage, cell source, and administration methods of hMSCs to promote blood flow regeneration and the functional recovery of affected tissues or organs to avoid fatal consequences. 

Recently, a gene therapy approach has also been explored, in which genetic material encoding for proteins that may help to increase revascularization is injected into patients, but it showed no clear differences between treatment groups [8].

The failure of these procedures may suggest that either MSC therapeutic mechanisms remain elusive or that the disease condition affects the results. Both of these require further investigation.

Tissue ischemia *per se* is not sufficient to regenerate vascular damage, although several studies demonstrated that ischemia is able to stimulate neoangiogenesis through HIF1-mediated processes [9], to induce the secretion of growth factors (i.e., VEGF) and cytokines from the surrounding cells [10], and to promote the recruitment of precursor cells to injured sites by the homing and migration of transplanted hMSCs to ischemic tissues in animal models [11].

The homing and migration of transplanted hMSCs are the major challenges to be solved in stem cell-based therapy. These processes have been compared to leukocytes due to their common features, such as cytokine responsiveness and ability for transendothelial migration [12,13]. A multitude of molecular signals and messengers play an essential role in leukocyte migration, and it is increasingly recognized that microRNAs are important regulators of cell migration [14]. Starting from this, we decided to investigate the miRNA profile in hMSCs.

miRNAs are short (18–25 nucleotides), highly conserved, non-coding RNAs involved in the regulation of gene expression by binding to mRNA, leading to mRNA degradation or the inhibition of protein synthesis [15]. They represent an important regulatory pathway controlling cell homeostasis, whose deregulation is associated with several vascular diseases, highlighting their importance in vascular development, post-natal and pathological angiogenesis [16]. For example, miR-221 is required in vascular development [17]. Endothelial-specific miR-17a inhibits angiogenesis in ischemic models [18], as well as the deregulation of miR-503 [19].

Increasing attention has been given to secreting miRNAs. Circulating miRNAs have been reported as novel diagnostic or therapeutic targets in several human diseases, based on the analysis of miRNAs isolated from the plasma of patients [20,21,22]. However, the use of plasma miRNAs faces technical limitations because of the extremely low concentrations of miRNAs freely circulating in the plasma, leaving the underlying molecular mechanisms regulated by miRNAs poorly understood. 

To this end, we assessed the miRNA expression profile of healthy and CLI-derived hMSC cells as a model of ischemic disorders. Multi-omics profiling revealed two miRNAs as important regulators of cell migration: miR-29b as a potential marker of the disease and miR-638 as a potential therapeutic target. 

To our knowledge, this study is the first to identify the co-regulatory network among the hypoxia-regulated genes and miRNAs in human mesenchymal stem cells (hMSCs), the most promising cell source for cell therapies in regenerative medicine.

## 2. Results

### 2.1. miRNA Profiling of hMSC Derived from CLI Patients

The main aim of this study was to identify the differential miRNA expression profile between healthy hMSC cells (CTRL) and those derived from CLI patients (CLI), trying to understand the defective molecular mechanisms underlying a pathological condition. We used hMSC cells for their involvement in reparative processes in vivo. The clinical characteristics of these cells are reported in Supplementary Table S1. 

A principal-component analysis (PCA) revealed that healthy hMSCs and CLI-derived hMSCs were clearly separated (Figure S1). We then performed statistical analysis to identify miRNAs that were differentially expressed using a paired *t*-test (Supplementary Table S2–S5), comparing cells cultivated either in standard oxygen conditions (20% O_2_) or in hypoxic (0.5% O_2_) and serum-starved (0.5% FBS) conditions, to mimic pathological ischemia (referred as hypoxia), in which a lack of oxygen and nutrients is present (FC ≥ ±2; FDR ≤ 0.05).

**Figure 1:**
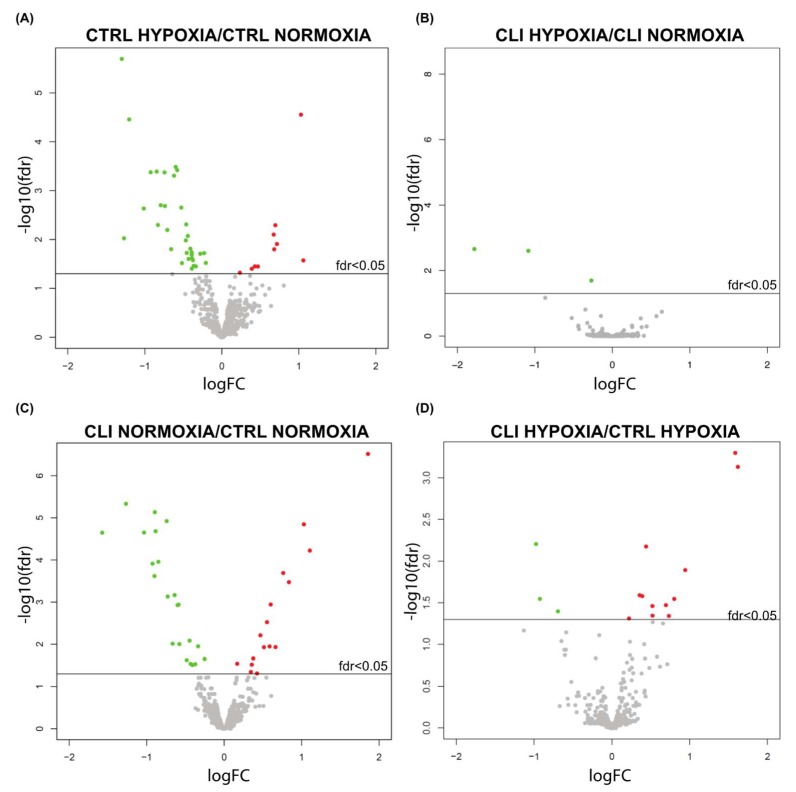
Volcano plots representing differentially expressed miRNAs (statistical significance vs. differential regulation) in our experimental settings. Significantly up- and down-regulated miRNAs (FDR <0.05) are displayed as red and green dots, respectively, and unchanged miRNAs as grey dots. (**A**) CTRL HYPOXIA/CTRL NORMOXIA; (**B**) CLI HYPOXIA/CLI NORMOXIA; (**C**) CLI NORMOXIA/CTRL NORMOXIA; (**D**) CLI HYPOXIA/CTRL HYPOXIA.

Figure 1 shows volcano-plots identifying changes in our data sets by plotting statistical significance versus differential regulation within our experimental settings. Significantly up- and down-regulated miRNAs (FDR < 0.05) are displayed as red and green dots, respectively, and unchanged miRNAs as grey dots.

**Figure 2:**
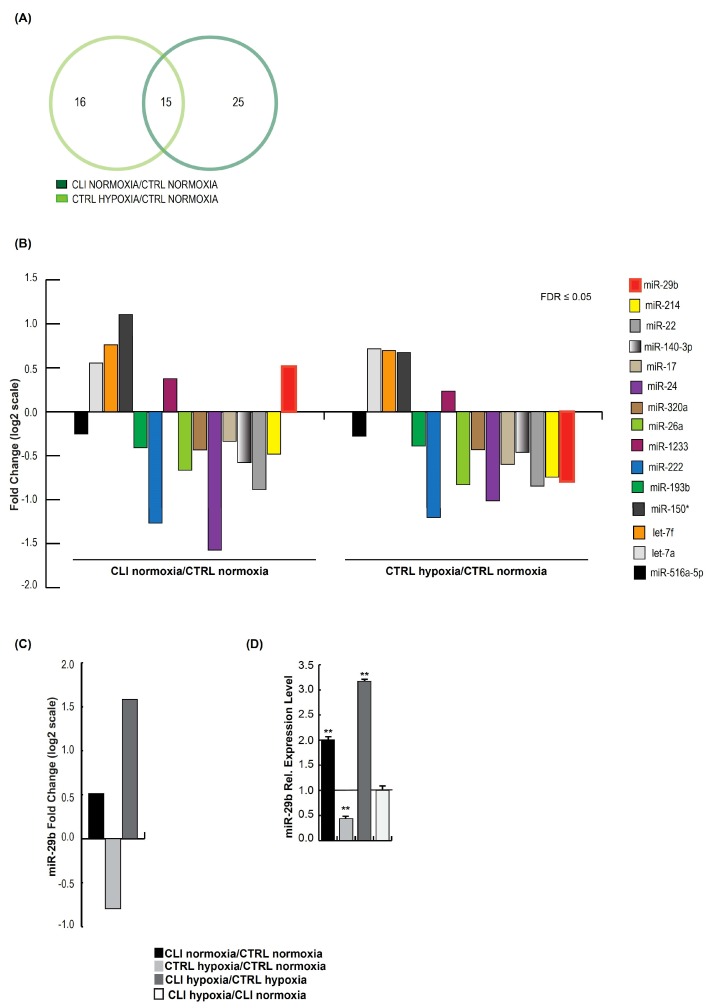
(**A**) Venn diagram showing 15 common miRNAs between CLI NORMOXIA/CTRL NORMOXIA vs CTRL HYPOXIA/CTRL NORMOXIA. (**B**) Fold change of microarray expression levels in log2 scale of 15 miRNAs commonly altered in CLI normoxia/CTRL normoxia vs. CTRL hypoxia/CTRL normoxia (*FDR* ≤ 0.05). (**C**) Fold change of microarray expression levels in log2 scale of miR-29b in CLI normoxia/CTRL normoxia, CTRL hypoxia/CTRL normoxia and CLI hypoxia/CTRL hypoxia. (**D**) Relative expression levels of miR-29 analysed by RT-PCR in CTRL HYPOXIA/CTRL normoxia, CLI normoxia/CTRL normoxia, CLI hypoxia/CTRL hypoxia and CLI hypoxia/CLI normoxia. Data are the result of three independent experiments each with three replicates, and are represented as ±SEM. RNU6B was used for data normalization for miRNA. *p*-value ≤ 0.01 (**).

On deeper analysis of the miRNA expression profiles, we identified a cluster of 15 common miRNAs modified in healthy hMSCs exposed to pathological conditions (0.5% O_2_ + 0.5% FBS) and in CLI-derived cells (Figure 2A). Interestingly, all 15 miRNAs showed the same expression trend, except miR-29b (Figure 2B), whose expression was upregulated in CLI-derived cells compared to CTRL, where it was downregulated when healthy hMSCs were exposed to pathological conditions (0.5% O_2_ + 0.5% FBS). A further upregulation of miR-29b (three-fold) was seen when CLI cells were exposed to 0.5% O_2_ + 0.5% FBS (Figure 2C). These results were validated by RT-PCR analysis in all experimental settings (Figure 2D) and it was noted that miR-29b is significantly upregulated in CLI cells, suggesting that miR-29b could be considered a pathological marker.

To identify the targets of miR-29b, we consulted miRSystem, a database that integrates seven well-known miRNA target gene prediction programs (DIANA, miRanda, miRBridge, PicTar, PITA, rna22, and TargetScan), and validated data from TarBase and miRecords.

A total of 907 predicted targets of miR-29b with HIT ≥ 3 criteria were found (Supplementary Table S6).

**Figure 3:**
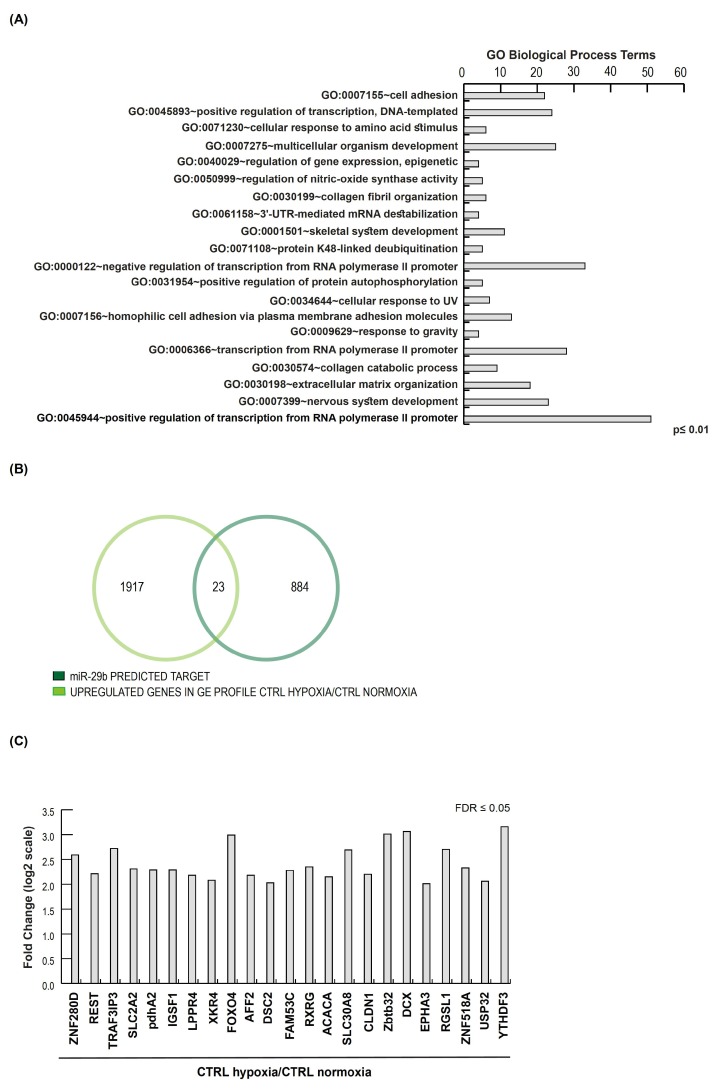
(**A**) GO term classification of 502 common hits intersecting the 907 predicted miR-29b target genes with 34017 experimentally defined regulated genes in GE microarray CTRL HYPOXIA/CTRL NORMOXIA (FDR ≤ 0.05 and FC ≥ 2). (**B**) Venn diagram showing 23 common genes between 907 predicted miR-29b target genes and 1940 upregulated genes in GE microarray CTRL HYPOXIA/CTRL NORMOXIA (FDR ≤ 0.05 and FC ≥ 4). (**C**) Fold change of microarray expression levels in log2 scale of 23 common genes.

To validate the miR-29b target prediction, we analyzed published GE profiles of human mesenchymal stem cells cultured in 0.5% and 21% O_2_ for 24 h by GEO2R (GSE55875) [23]. Intersecting the 907 predicted miR-29b target genes with 34017 experimentally defined regulated genes in the GE microarray, we found 502 common genes that were actively involved in the cell adhesion process (*p*-value <0.01), showing an enrichment in the ECM–receptor interaction pathway (*p*-value < 8.6 × 10^−5^), and in the collagen and cadherin protein domain (*p*-value < 6.9 × 10^−6^) in stemness (Figure 3A, Supplementary Tables S7 and S8).

According to miR-29b downregulation, we selected 1940 upregulated genes in CTRL cells in the hypoxia condition (FC LOG2 ≥ +2). Comparing them with 907 predicted miR-29b target genes, we identified 23 gene hits showing a direct inverse correlation with miR-29b expression (Figure 3B,C). Interestingly, the functional annotation of these 23 genes by DAVID Bioinformatics Resources 6.8 showed their critical role in cellular responses to hypoxia, specifically in pathways for which changes in the oxygen concentration were transduced in gene expression modifications (pathways: glucagon signaling pathway and pyruvate metabolism, Supplementary Table S9). In addition, many genes are transcription factors that regulate cell adhesion and migration [24,25,26]

**Figure 4:**
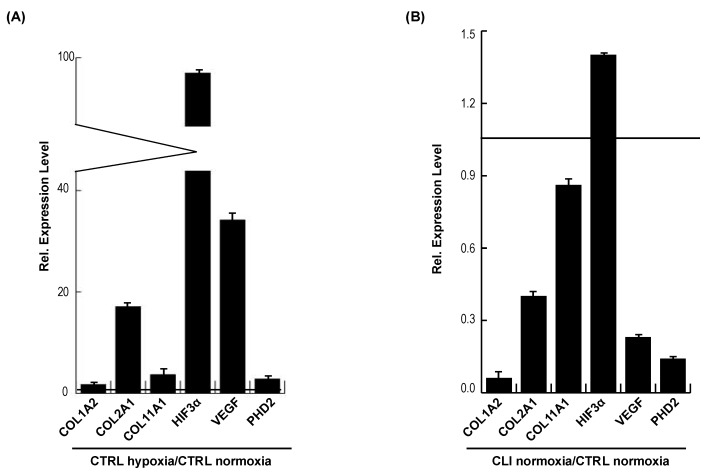

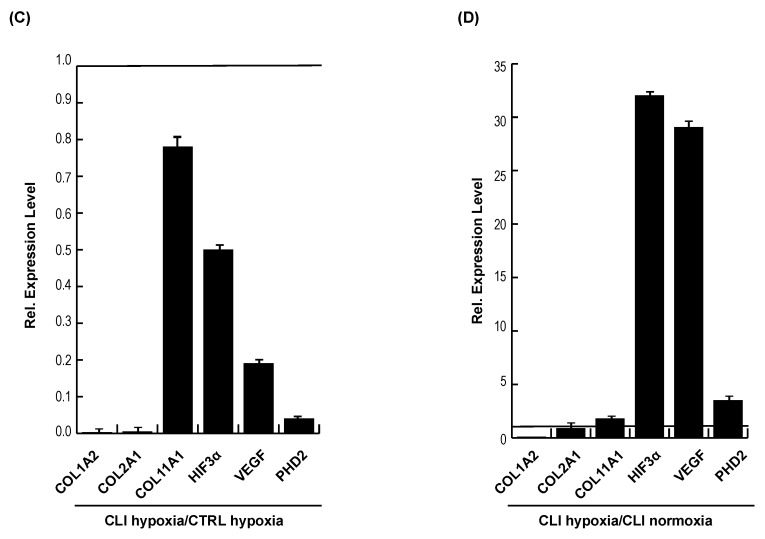
Relative expression levels of miR-29b targets analyzed by RT-PCR in CTRL hypoxia/CTRL normoxia (**A**), CLI normoxia/CTRL normoxia (**B**), CLI hypoxia/CTRL hypoxia (**C**) and CLI hypoxia/CLI normoxia (**D**). Data are the result of three independent experiments each with three replicates, and are represented as ±SEM. EAR was used for data normalization for gene expression. *p*-value ≤ 0.01.

Altogether, these data hint at a direct role of miR-29b in the regulation of hypoxic gene expression, specifically in the pathological condition and cell migration. Among the retrieved putative targets, we looked for genes belonging to these pathways (COL1A2, COL2A1, COL11A1, HIF3A, VEGF, PHD2), and we compared and contrasted the expression of genes in healthy and CLI cells. We validated miR-29b predicted targets by quantitative real-time PCR (Figure 4A–D). RT-PCR showed a drastic reduction in all genes analyzed in CLI cells compared to CTRL cells, so that the hypoxia signaling and collagen production were defective, suggesting that miR-29b could be considered a novel pathological biomarker (Figure 4A–D).

### 2.2. microRNA 638 (miR-638) as a Potential Therapeutic Target 

**Figure 5:**
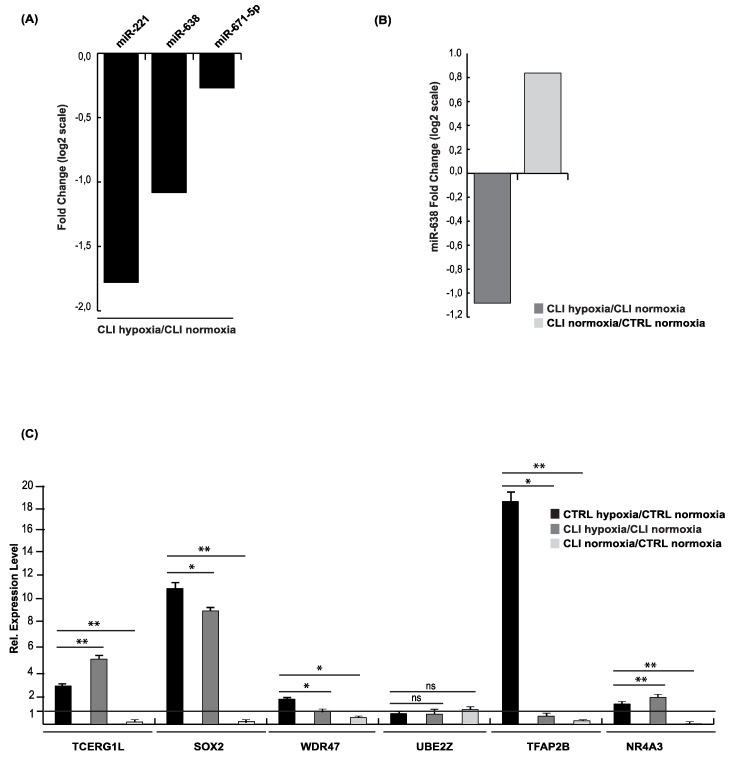
(**A**) Fold change of microarray expression levels in log2 scale of miR-638, miR-221 and miR-671-5p in CLI hypoxia compared to CLI normoxia. (**B**) miR-638 expression in CLI hypoxia compared to CLI normoxia and in CLI normoxia compared to CTRL normoxia. (**C**) Relative expression of miR-638 target genes by RT-PCR. Data are the result of three independent experiments each with three replicates, and are represented as ±SEM. RNU6B was used for data normalization for miRNA. *p*-value ≤ 0.05 (*); ≤ 0.01 (**).

On analysis of the miRNA expression profiles of CLI-derived cells exposed to hypoxia (CLI HYPOXIA) compared to normoxia (CLI NORMOXIA) cells, we found three differentially expressed miRNAs (Figure 5A) (FC ≥ ±2; FDR ≤ 0.05). Among these, hsa-miR-221 has already been reported to be involved in normal vascular development, regulating EC sprouting and migration [17,27]. hsa-miR-671-5p is a tumor-suppressor miRNA that is significantly decreased in invasive breast cancer by the deregulation of FOXM1, promoting cell invasion [28]. Indeed, hsa-miR-638 is involved in cell migration, targeting Nor1 [29]. All of these seem to be implicated in the migration process. Of these, we focused on miR-638 which is, in contrast, upregulated in CLI in normoxia conditions (Figure 5B). This upregulation seems to be restored at the CTRL level when CLI cells are cultured in hypoxia. *De facto*, miR-638 is upregulated in CLI NORMOXIA cells (FC = 0.836), but the hypoxia condition restores the expression to the CTRL NORMOXIA level (CLI HYPOXIA/CLI NORMOXIA FC = −1.08).

This peculiar expression was allowed to proceed to target prediction using the miRSystem database (HIT≥3) and GE comparison, as previously described. In Supplementary Table S10, we list the miR-638 predicted target genes that were actually altered in the GE profiles. The expression level of the upregulated targets was verified by quantitative RT-PCR (Figure 5C). Many of them are regulated by hypoxia and hence may serve as potential targets for improving therapeutic use, although further analysis will be required.

These findings suggest that miR-638 could be a therapeutic miRNA, providing new insights into the understanding of the pathogenesis of CLI, but also suggesting novel, putative targets for therapeutic approaches.

## 3. Discussion

Based on multiple *in vitro* and pre-clinical studies, MSC-based therapy has reached the bedside as a new therapeutic option to treat severe diseases in clinics. Since 2010, more than 50 clinical trials have been conducted using MSC cell therapy due to its multilineage differentiation capacity, immunomodulatory and anti-inflammatory effects, homing and migratory capacity to injured sites. However, the efficiency of MSC therapy is rather low, an there is insufficient evidence to support cell therapy in clinical practice [3,30,31].

To date, the mechanisms by which autologous transplantation improves clinical outcomes in patients with ischemic disorders such as CLI indicate that hMSCs have the capacity to stimulate the formation of new blood vessels either by stimulating endothelial cell proliferation and migration [32], by secreting soluble factors able to stimulate the sprouting of endothelial cells [33,34], as well as by providing precursor cells able to integrate into vascular beds [35].

The combination of these mechanisms led to the amelioration of tissue perfusion, but limited results were observed in clinical trials due to poor cell engraftment, short cell persistence after transplantation, and a low survival rate [36], all processes based on cell homing and engraftment.

In this study, we used an -omics approach to identify deregulated miRNAs in hMSC cells exposed to conditions mimicking the pathological feature of ischemic disorders i.e., lack of oxygen and nutrients. 

As a result of our analysis, we retrieved miR-29b as a potential pathological marker of ischemia, due to the negative effect on the hypoxia signaling genes and ECM protein synthesis when upregulated. 

HIF3A, VEGF and PHD2 are all involved in hypoxic response [37]. We recently reported that HIF3A is upregulated in hMSC cells, both in normoxia and hypoxia, and its regulation is driven by different cytokines. In particular, when hMSCs are exposed to VEGF, the accumulation of HIF3A is more evident, suggesting a role in promoting angiogenesis [38].

Published data has already reported that the upregulation of miR-29b has been involved in inhibiting angiogenesis. The ectopic expression of miR-29b inhibits HUVEC cells from forming three-dimensional capillary-like tubular structures, cell proliferation and migration [39]. In addition, the systemic administration of miR-29b potently suppresses tumor vascularization by targeting VEGF-A, resulting in the dramatic suppression of tumor growth without toxicity, indicating that this single miRNA could be used as an efficient anti-cancer therapeutic agent [39]. 

By bioinformatic and RT-PCR analysis, we revealed that miR-29b could regulate the expression levels of metalloproteinase (MMP-2 and MMP-8) and collagen genes (COL1A2, COL2A1, COL11A1). The MMP enzymes are a family of zinc-dependent endopeptidases that degrade various proteins in the extracellular matrix (ECM), including collagen, and alteration of this process could influence vascular remodeling [40].

Published in vitro studies showed that the overexpression of miR-29b significantly decreased the mRNA and protein level of MMP2 and the activity of MMP2 to suppress gastric cancer cell migration [41].

Therefore, our analysis suggests that the possible failure of vascular remodeling observed in CLI patients could be mediated in part by the down-regulation of genes involved in hypoxia signaling (VEGF, HIF3A, PHD2) and ECM protein synthesis (COL1A2, COL2A1, MMP2) via miR-29b [42]. In addition, the migration of hMSC cells toward injured sites may be affected by a lack of MMP2, which affects their homing [43].

On looking for a therapeutic marker, we found that miR-638 expression showed an inverse correlation comparing the normal and pathological conditions. It has been reported to be involved in MSC functions such as cellular proliferation, differentiation, migration, and cell death [44]. Decreased levels of miR-638 in serum were associated with an increased risk of liver metastasis and the later TNM stage of colorectal cancer [45], as well as in breast cancer [46]. miR-638 is a key molecule in regulating human vascular smooth cell proliferation and migration by targeting the NOR1/cyclin D pathway and specific modulation of miR-638 may represent an attractive approach for the treatment of vascular diseases [29].

Therefore, our analysis reveals that the down-regulation of miR-638 occurring in hypoxia could affect cell migration.

Although preclinical studies have enlarged our knowledge of the pathophysiological mechanisms of ischemic diseases, there is still no validated therapy to successfully treat ischemic tissues. Human MSCs, with their fascinating properties, are used in clinical trials, but we still do not know how to make them more efficient.

Here, we used a computational approach starting from miRNA expression in hMSCs to identify other targets that could be modulated to achieve a therapeutic goal.

We believe that our study provides significant novel insights and potential targets for the prevention and treatment of human vascular diseases. 

## 4. Methods

### 4.1. Isolation of Human BMSCs (hBMSCs)

Human mesenchymal stem cells (hMSC) were isolated from patients with end-stage CLI (*n* = 2) (III or IV stage of Leriche–Fontaine classification) enrolled for treatment with autologous bone marrow cells transplantation between February 2008 and July 2008. The protocol was approved by the Institutional Ethics Committee of Universita’ L. Vanvitelli of Naples and was registered at the Trial Registration site, NCT00306085. All patients gave written informed consent before evaluation for inclusion in the study (Supplementary Table S1).

For healthy controls (*n* = 2), we collected bone marrow aspirate removed from a male who had undergone orthopedic surgery (Ctr#1) which would normally have been discarded, and the other hMSC cell line was purchased from ProVitro AG (Ctr#2: ProVitro AG, Berlin, Germany #1210911).

### 4.2. Human Bone Marrow Stem Cell Harvesting

A fraction of the 10 mL bone marrow aspirates from the CLI patients subjected to autologous transplantation [33,47] was collected in tubes with NaCitrate (129 mM) and then diluted with PBS (1:3) and mononuclear cells isolated by density gradient centrifugation with Ficoll. Mononuclear cells were washed with PBS (3×) and plated in DMEM medium (Corning, 10-013-CM, New York, NY, USA), 1% antibiotics-antimycotics (Invitrogen, 15240-062, Carlsbad, CA, USA) and 10% fetal bovine serum (FBS), into 100 mm culture dish and incubated at 37 °C in a humidified atmosphere at 5% carbon dioxide (CO_2_). Non-adherent cells were removed after 24 h, the adherent cells carefully washed in phosphate-buffer saline (PBS) and further expanded in fresh culture medium. Culture-expanded BMSCs of passage 2–5 were used for miRNA array and quantitative real-time polymerase chain reaction (qPCR). Hypoxic culture conditions were obtained by BD GasPak EZ Anaerobe Gas Generating Pouch System (BD Biosciences, San Diego). As certified by the manufacturer, the Anaerobe Gas Generating Pouch System produces an atmosphere containing 10% carbon dioxide and 1% oxygen. Confluent healthy and CLI hMSC monolayers were subjected to normoxic and hypoxic culture for 24 h. Cells were serum-starved in EBM plus 0.5% fetal bovine serum (FBS) at least 8 h before hypoxic culture to minimize the effects of growth factors in the expansion media.

### 4.3. RNA Isolation and miRNA Expression Analysis

Total RNA, miRNA enriched, were isolated and miRNA expression levels analyzed by real-time PCR as described [48].

### 4.4. Real-Time PCR

Real-time PCR was performed using RNA VILO cDNA Synthesis Kit (Invitrogen, Carlsbad, USA), to convert RNA into cDNA. For a total of 50 ng of cDNA, a 1xSybrgreen PCR Master Mix (BioRad, Hercules, USA) was used according to the supplier’s instructions. Primers are listed in Supplementary Table S9.

### 4.5. miRNA Microarray Profiling and Data Analysis

miRNoma of the CLI-derived and control hMSCs in hypoxia and normoxia condition were analyzed. Each sample was prepared according to the Agilent’s miRNA Microarray System protocol. Total RNA (100 ng) was dephosphorylated with calf intestine alkaline phosphatase (GE Healthcare Europe GmbH, Little Chalfont, England), denatured with DMSO (Sigma), and labelled with Cyanine 3-pCp by T4 RNA ligase (GE Healthcare Europe GmbH). The labelled RNAs were purified and then hybridized to human miRNA Microarray (V1)8x15K (Agilent Technologies, G4470A, Santa Clara, USA) for 20 h at 55 °C with rotation. After hybridization and washing, the arrays were acquired with an Agilent Scanner and data extracted using Agilent Feature Extraction Software, as specified by the manufacturer (Agilent Technologies, Santa Clara, CA, USA).

Microarray quality control reports generated by the Agilent Feature Extraction software were used to detect hybridization artefacts. Probe level raw intensity were processed using R/Bio Conductor [49] and Limma package. Background correction was performed using “normexp” limma method, and data normalization was carried out in two steps: less normalization within-array to correct systematic dye-bias; and quantile normalization between–arrays to detect systematic non-biological bias. Ratios representing the relative target mRNA intensities compared to control RNA probe signals were derived from normalized data. In order to detect the statistical significance of differential expression, we performed Student’s *t*-test. For each *p*-value, the Benjamini–Hochberg procedure was used to calculate the false discovery rate (FDR) to avoid the problem of multiple testing. The selected miRNA list was obtained using the following thresholds: FDR < 0.05 and abs(ratio) > 2; each value was converted in LOG2. 

Microarray data were deposited in the Gene Expression Omnibus (GEO) database (http://www.ncbi.nlm.nih.gov/gds) under the accession number: GSE125884.

### 4.6. Computational Prediction of miRNA Target Genes

Target gene prediction of the differentially expressed miRNAs was performed using the miRSystem database. Target genes were selected by the application of HIT ≥3 or if they are present in at least 3 of miRNA target gene prediction programs, then DIANA, miRanda, miRBridge, PicTar, PITA, rna22, and TargetScan were used; and in validated data from TarBase and miRecords.

### 4.7. Gene-Enrichment and Functional Annotation Analysis

The relative abundance of “biological process” (BP), pathways (KEGG) and protein interactions (INTERPRO), and gene ontology terms in each of the selected lists was analyzed using the Database for Annotation, Visualization and Integrated Discovery (DAVID) Functional Annotation Clustering tool [50].

## Supplementary Materials

Supplementary materials can be found at https://www.mdpi.com/1422-0067/20/6/1340/s1.
